# Epicutaneous immunotherapy in rhino-conjunctivitis and food allergies: a review of the literature

**DOI:** 10.1186/s12967-018-1701-6

**Published:** 2018-11-27

**Authors:** Susanna Esposito, Chiara Isidori, Alessandra Pacitto, Cristina Salvatori, Laura Sensi, Franco Frati, Giuseppe Di Cara, Francesco Marcucci

**Affiliations:** Pediatric Clinic, Department of Surgical and Biomedical Sciences, Università degli Studi di Perugia, Piazza Menghini 1, 06129 Perugia, Italy

**Keywords:** Allergen immunotherapy, Epicutaneous immunotherapy, Food allergy, Allergic rhinitis, Tape stripping

## Abstract

**Background:**

Epicutaneous immunotherapy (EPIT) is a new way of allergen administration that has a high rate of adherence and safety. The aim of this manuscript is to review clinical trials on EPIT for respiratory and food allergies published in the last 10 years, taking into account how different variables (i.e., dose, patch application duration, skin preparation, and efficacy and safety evaluation) have influenced study results.

**Main body:**

From a review of the literature, we identified eight placebo-controlled, double-blind trials conducted on children and adults, including four studies on grass pollen rhino-conjunctivitis, one on cow’s milk allergy and three on peanut allergy. Different methods for skin pre-treatment, such as skin abrasion and tape stripping or stratum corneous hydration by an occlusive system, different endpoints and cumulative allergen doses, and different durations of patch application and tape stripping, were used in the rhino-conjunctivitis studies. A visual analogue system was used for the efficacy evaluation. Several local skin reactions (eczema) and some systemic adverse reactions were reported at higher rates in the active group compared to placebo in one study, but this was not shown by other authors. Local eczema reactions were correlated to the times for applying the tape stripping, while systemic side effects were correlated to the deepness of scraping. In the food allergy trials, differences in the food challenge thresholds, endpoints and allergen sites of the cutaneous patch application influenced the study results. A slight dose-dependent efficacy was found in the peanut allergy studies, which was confirmed by a more significant increase in the following progressive open study. Few adverse events and high adherence in all of the food allergen trials were reported.

**Conclusions:**

Overall, the EPIT study results, even if they were affected by great heterogeneity among the methodologies applied, have shown not only the high safety and adherence with this kind of immunotherapy but also suggested the possibility for obtaining definitive evidence of the efficacy of EPIT, especially for food allergies.

## Background

In industrialized countries, allergic rhinitis (AR) and food allergies (FA), which are highly prevalent at 40% and 8%, respectively, are considered emerging problems of public health because of their correlation with a reduction in quality of life and medication abuse [1–3]. As recommended by the guidelines of the European Academy of Allergy and Clinical Immunology (EAACI), allergen immunotherapy (AIT) aims to gain long-term clinical benefit by changing the natural history of allergic disease [4]. The first AIT administration route, which was identified approximately a century ago, was subcutaneous immunotherapy (SCIT) followed by sublingual immunotherapy (SLIT) [4–6]. The first study on epicutaneous immunotherapy (EPIT) was reported in 1921 by Valery-Radot et al. [7], who showed that allergen administration on scarified skin reduced allergic symptoms in patients allergic to horses. In the following years, the results of its safety and efficacy obtained by Blamontier et al. [8, 9] with the “quadrillage” method of skin scarification opened the way to several French and other European authors, who obtained further evidence of the potential of this procedure [10–12]. After these encouraging studies and with the aim to find an administration route safer than SCIT and obtain more adherence than SLIT, Senti et al. [13], with their first double-blind placebo controlled EPIT study, elected the epidermidis as a new promising route for specific immunotherapy.

Two different epicutaneous delivery systems were developed to improve allergen skin penetration: adhesive tape stripping and the hydration/occlusive system [13, 14]. Tape stripping consists of the physical removal of the epidermis corneal layers before allergen patch administration. This process increases allergen passive diffusion through the epidermal layers and creates a pro-inflammatory state that enhances the interaction between the allergen and antigen presenting cells (APCs) [15]. The other method consists of an occlusive chamber permitting water going up from the basal layer to the corneum stratum of the epidermidis [15]. Through dilated intercellular spaces, the allergen can reach the deep epidermidis strata, where it is captured by Langerhans cells and transported to loco-regional lymph nodes [15]. Kinetic studies in murine models showed that dendritic cells migrate in the draining lymph node after 6 h from patch application on intact skin, reaching their highest concentration after 24 h and then slowly decreasing [16]. The aim of this manuscript is to review EPIT clinical trials on respiratory and food allergies published within the last 10 years. Their safety and efficacy have been evaluated in relation to the different variables among the studies, and their influence on the reliability of the results have been analysed.

## Epicutaneous immunotherapy (EPIT) for aeroallergens

### Available studies

The available studies on EPIT for aeroallergens are summarized in Table 1. All the studies were performed with Phl p 5.

**Table 1:** Summary of the epicutaneous immunotherapy (EPIT) aeroallergen clinical trials

References	Study population	Single-patch allergen dose	Cumulative-patch allergen dose	Skin preparation methods	Patch application time (h)	Primary outcomes	Secondary outcomes	Results with statistical and/or clinical significance: placebo vs. verum
Proof of concept trial: Senti et al. [13]	37 people	21 μg	252 μg	Tape-stripping	48	NPT	SS MS AEs	SS AEs
Efficacy trial: Agostinis et al. [17]	30 children	11.25 μg	135 μg	Idratation	24	SPT SS MS	None	SS MS
Dose-ranging trial: Senti et al. [18]	132 people	3 μg 15 μg 30 μg	18 μg 90 μg 180 μg	Tape-stripping	8	SS	wSS MS CPT SPT	SS^a^ wSS^b^
Immune-response trial: Senti et al. [15]	98 people	21 μg	126 μg	Tape-stripping Abrasion	8	SS	MS CPT IgG4 IgE	SS IgG4^c^

All the EPIT clinical studies were performed with Phl p 5

*NPT* nasal provocation test, *SS* symptoms score, *MS* rescue medication score, *AEs* adverse events, *SPT* skin prick test, *wSS* weekly recorded symptoms, *CPT* conjunctival provocatin test

^a^Placebo vs. high-dose verum group in the follow-up period

^b^Placebo vs. high-dose verum group both in the treatment period and in the follow-up period

^c^Placebo vs. treated group in the treatment period

The first pilot EPIT study by Senti et al. [13] was a monocentric, placebo-controlled, double-blind trial conducted to assess the efficacy and safety of EPIT in respiratory allergy patients. Thirty-seven adult patients were enrolled and randomized to receive patches containing allergen or placebo. The patch was applied weekly for 12 weeks and left on for 48 h on skin that was stripped six times. The single patch dose was 21 μg of Phl p 5 (Phelum P.), while the cumulative dose was 252 μg. Changes in the nasal provocation test (NPT) in the treated group compared to placebo were considered the primary outcome. The secondary outcomes were symptoms scored using the visual analog scale (VAS), the use of rescue medications recorded daily in a personal diary, and the occurrence of local reactions and systemic adverse events. Statistical significance was reached only for the symptoms evaluated by the VAS scale. The results regarding the safety and tolerability showed a significant difference in adverse local and systemic reactions that were mostly reported in the allergen-treated group. No severe systemic allergic reactions were described [13].

Agostinis et al. [17] conducted a prospective double-blind randomized trial on 30 children with seasonal rhino-conjunctivitis, which had the aim of preventing respiratory symptoms. The single patch dose was 11.25 μg of Phl p 5, while the cumulative dose was 135 μg, which was distributed in 12 patches. Patches were applied weekly for 12 weeks, and they were maintained on the patient’s back for 24 h. The primary outcome measures were the skin prick test (SPT), daily rhino-conjunctivitis symptoms and assumption of antihistamine drugs. No statistically significant differences in the SPT were found between the treated and placebo groups. There was a statistically significant improvement in rhinorrhoea, nasal obstruction, dyspnoea and ocular tearing in the treated group compared to the placebo group. In the active group, ocular itching increased significantly. There were no systemic reactions and no local adverse events. A statistically significant reduction in the use of antihistamine drugs was recorded in the treated group.

The second study by Senti et al. [18] was a dose-escalation trial that had the aim of optimizing the treatment dose of EPIT in grass pollen rhino-conjunctivitis. One hundred thirty-two adult patients were enrolled and randomized in four groups: three received different treatment doses, and one received placebo. Six patches were applied for a period of 8 h on skin that was tape stripped six times. The cumulative doses were 18 μg, 90 μg and 180 μg of Phl p 5 for the low, medium and high dose groups, respectively. The subjective symptom score was considered the primary endpoint and was evaluated through a VAS scale. Statistically significant differences were found in only the high-dose treated patients during the follow-up period. The conjunctival provocation test (CPT) and medication scores were also evaluated but were not significantly different. Local adverse events were more frequent in patients treated with higher doses and decreased with the following patch application. Systemic adverse events treated with intravenous antihistamines and corticosteroids were observed in 8.3% of the patients.

A third trial by Senti et al. [19] was presented as a summary of three clinical trials of these authors. In this study, specific immunological changes after allergen-specific immunotherapy were evaluated. It was a monocentric, placebo-controlled, double-blind phase I/IIa trial that included 98 patients randomized to receive placebo or allergen. Six patches were applied weekly and left on the upper arm for 8 h. The skin was pre-treated by abrasion for the first 52 patients and by tape stripping for the other patients. The single and cumulative patch doses of Phl p 5 were 21 μg and 126 μg, respectively. The symptoms were scored by a VAS scale and were used as the primary outcome. They reached statistically significant improvements in the treated group. The secondary outcomes were the medication score, CPT, and allergen-specific IgG4 and IgE. A statistically significant reduction in conjunctival reactivity in the treated group was found. Allergen-specific IgG4 reached a statistically significant increase in the treated group only in the first pollen season, while allergen-specific IgE did not show any variation. Local adverse events (18%) were correlated with the duration of patch application. Systemic reactions occurred in seven of the treated patients, and they were correlated with the degree of stratum corneum disruption.

### Critical analysis of the results

In their three studies [13, 18, 19], Senti et al. used different methods at the aim to improve the safety and efficacy of treatment, taking into account the data obtained from the first trial. In fact, in their first study, the authors showed, by a VAS scale, that EPIT may induce a very significant improvement of symptoms (> 70%) in treated patients, but it was paralleled by a significant increase in local adverse effects compared to placebo.

With the purpose of reducing local adverse reactions, in the second trial by Senti et al. [18] the patients were treated with three different doses of allergens. They reduced the number of patch applications from 12 to 6 and reduced the duration of patch application from 48 to 8 h. In this dose-escalation study, they obtained a significant improvement of symptoms (70%) with the higher dose of Phl p 5 (30 µg), which was similar to the results obtained with 21 µg of the same allergen in the previous study [13]. They also showed a relevant reduction of local adverse reactions, but unfortunately 10 systemic adverse events that were not previously evidenced were observed.

In the third study [19], the authors maintained the reduction of the duration and number of patch applications, but due to the systemic adverse effects observed in the second study [18], a lower allergen dose was given compared to that given in the second study but similar to that employed in the first study (21 µg). At the same time, they increased the number of skin pre-treatments from 6 to 10, which were performed with two different methods, abrasion and tape stripping. In this last trial, they confirmed the efficacy of EPIT but observe a reduction in the efficacy from 70 to 48% in comparison with the previous works. Local adverse effects were again reduced, but systemic effects persisted with the tape-stripping method and were more frequent with the abrasion method.

Agostini’s study [17] performed on intact skin with hydration by an occlusive chamber also further confirmed the efficacy of EPIT in children affected by grass pollen rhino-conjunctivitis without raising local or systemic adverse events. Although the dose of 11.25 µg of Phl p 5 seemed effective in symptoms score and rescue medication score, it was lower than the one recommended in Senti’s studies [13, 15, 18].

The results of Senti’s studies suggest that the EPIT efficacy is dose-dependent. It is likely that the single dose adequate for the type of skin pre-treatment still has not been found. It seems also probable that similar to SCIT and SLIT, the cumulative dose might be significantly raised, increasing the number of patch applications for an effective long-lasting effect. Adverse local reactions are not severe, even if they are much more frequent, while adverse systemic reactions were not severe (0.4–0.6%) and never needed epinephrine treatment. Tape stripping corneum stratum disruption may be the main event correlated with the adverse reaction observed, even if other variables may contribute to it, like specific constitutional skin determinants due to age, gender, race and others [20].

In conclusion, these first studies on patients with grass pollen rhino-conjunctivitis, even though they were performed without strictly comparable methods and were quite heterogeneous, confirm that EPIT is effective and safe [13, 15, 17, 18].

However, further studies performed in accordance with European and international guidelines are needed to definitively confirm these exiting results.

## Epicutaneous immunotherapy (EPIT) in food allergy

### Available studies

The available studies on EPIT for FA are summarized in Table 2. All the studies were performed with whole extract.

**Table 2:** Summary of the epicutaneous immunotherapy (EPIT) for food allergy clinical trials

References	Study population	Allergen	Single-patch allergen dose	Patch application time (h)	Safety	Efficacy	
						Primary outcomes	Results with statistical/clinical significance
Proof of concept trial: Dupont et al. [21]	19 children	Cow’s milk	1 mg	48	Local AE: TG: 4 subjects PG: 2 subjects Systemic AE: TG: 24 times PG: 8 times	OFC: increase in cumulative tolerate dose from baseline (< 10 ml) after 3 months	None
Safety and tolerability trial: Jones et al. [22]	100 mixed population^a^	Peanuts	20 μg 100 μg 250 μg 500 μg	24 48	TEAE: TG: 52.5% PG: 45% L-TEAEs: TG: 84% PG: 64%		
Efficacy, safety and dose-ranging trial: Sampson et al. [23]	221 mixed population^a^	Peanuts	50 μg 100 μg 250 μg	24	TEAE: 50 μg: 96.2% 100 μg: 94.6% 250 μg: 96.4% PG: 48.2% 1 moderate anaphylaxis	OFC after 12 months (1000 mg or tenfold increase)^b^	Overall response rate 250 μg-placebo: 50–25% (p = 0.01) 6–11 year stratum 250 μg placebo: 53.6–19.4% (p = 0.008)
	171 mixed population	Peanuts	50 μg 100 μg 250 μg	24	TEAEs: 93% in year 1 of extension 62% in year 2 of extension	OFC after 12 and 24 months	Overall response rate 12 months: 59.7% 24 months: 64.5%
Efficacy, safety and immune-response trial: Jones et al. [24]	74 mixed population^c^	Peanuts	100 μg 250 μg	24	Patch site AE: 100 μg: 79.8% 250 μg: 79.8% PG: 14.4% Non-patch site AE: 100 μg: 0.2% 250 μg: 0.1% PG: 0.2% 1 systemic hives	OFC after 52 weeks (5044 mg or tenfold increase)^d^	100 μg-placebo: 45.8% (p = 0.005) 250 μg-placebo: 48% (p = 0.003)

All the studies were performed with whole extract

*EDS* epicutaneous delivery system, *OFC* oral food challenge, *TEAEs* adverse events, *L-TEAEs* local adverse events

^a^Children and adults

^b^Changes in the OFC from baseline

^c^Comparison between 100 μg and 250 μg-dose group

^d^Expressed as a percentage

There is a particular interest in the development of new food allergy immunotherapy routes for the lack of successful desensitization procedures in this field [3]. Recently, four double-blind, placebo-controlled EPIT trials have been performed using the Viaskin patch system on intact skin. The evaluation of the safety and efficacy by EPIT was performed by an oral food challenge (OFC) to define the threshold of reaction to the allergen at baseline and after treatment, according to the EAACI guidelines [3].

The first trial [21] tested EPIT safety and tolerability in 19 children with a history of cow milk allergy. The cumulative dose of skimmed cow’s milk powder (36 mg) was administered for 3 months. The results of this study showed an increase in the cumulative tolerated dose (CTD) in the treated group, although the comparison with placebo did not reach statistical significance. The results of the epicutaneous delivery system were that it was well-tolerated and without severe adverse events. Local reactions were more frequent, but not significantly more frequent, in the active group. Only gastrointestinal side effects were significantly more frequent in the active group compared to the placebo group (16/470 vs. 0/316, p < 0.001).

A second trial [22] was performed to assess the clinical safety and tolerability of EPIT in peanut allergies. One hundred patients aged from 6 to 50 years with peanut allergies were randomized to four dosing cohorts, which received 20 μg, 100 μg, 250 μg, or 500 μg of peanut protein. The patches were applied for 24 or 48 h, and the entire treatment was 2 weeks long. In this study, EPIT by Viaskin resulted in being safe and well-tolerated in all of the ages. No significant difference in systemic adverse events versus the placebo group were reported. Only three subjects in the treated group dropped out. In addition, in the 24 h regimen, fewer local adverse events were reported compared with the 48 h regimen. There were no differences in the safety profiles of the different ages or in the cohorts with different peanut allergy severities. The IgE levels and SPT did not change at the end of the study. The efficacy was not evaluated.

In the third double-blind study [23], 221 people with peanut allergies, aged from 6 to 55 years, received different doses of allergen (50 μg, 100 μg or 250 μg) using Viaskin patches, which were applied daily for 12 months. For the patients < 12 years of age, it was applied to the upper central back area, and for those 12 years of age and older, it was applied to the inside of the upper arm. The food challenge-eliciting dose at baseline was equal to or inferior to 300 mg of peanut protein. The primary endpoints were the tolerance of 1000 mg or more at 52 weeks or a tenfold increase in the food challenge dose compared to baseline. At 12 months, a significant difference in the response rates was observed only between the high dose group and the placebo group (50% vs. 25%, p = 0.01). Additionally, the patient subgroup of age < 12 years treated with the same dose (250 mg) showed a more significant response rate compared to that of the placebo group (53.6% vs. 19.4%, p = 0.008). The median cumulative dose increased from 30 mg to 400 mg of peanut protein. The increase in specific IgE in the first 3–6 months was followed by a decrease at 12 months and by a fivefold increase in peanut IgG4 in all of the active treated groups. Mild or moderate local skin reactions were the most frequently occurring adverse event. Systemic severe adverse events were not reported. Adverse reactions were recorded in 48.2% of patients in the placebo group, while they were two times more frequent in the treated group (94.6–96.4%). After the first year, in an open-label extension period, 171 subjects were enrolled and randomized to initially receive patches with 50 μg, 100 μg or 250 μg of peanut, and after 6 months, all of them were switched to a 250 μg treatment. Food challenge, which was performed in 171/221 of the patients, showed an increase in the response rate from 59.7% at month 12 to 64.5% at month 24 (in patients of age < 12 years, it was from 63.3 to 68.4%). The mean cumulative reactive dose (CRD) increased from 44 to 440 mg, 1440 mg and 1440 mg after 1, 2 and 3 years, respectively, and 61% of the children treated with 250 µg achieved a CRD > 1000 mg. A reduction of adverse events to 62%, compared to 96% in the first year, was observed.

A last double-blind, placebo-controlled, multicentre trial [24] was performed in 74 people with peanut allergy aged 4–25 years. The treated group received 100 μg or 250 μg of peanut for a period of 52 weeks. In this study, the eligible dose of the double-blind, placebo-controlled challenge was less than or equal to 1044 mg, and the primary endpoint was a 5044 mg or a tenfold increase from baseline at 52 weeks. A significant dose increase was observed in 45.8% and 48% of the patients treated with the 100 µg and 250 µg doses, respectively (p = 0.005 and p = 0.003), which is compared to 12% in the placebo group. As for safety, local and systemic reactions occurred more frequently in the treated group, but this was without a statistically significant difference from the placebo group.

### Critical analysis of the results

In the cow milk pilot study by Dupon et al. [21], a suggestive increase in tolerance in ten treated children was reported, but this increase was not significant compared to the placebo. The low number of patients and low cumulative dose (36 mg) given for only 3 months may be responsible for this nonsignificant result. Local adverse events were more frequent in patients treated with cow milk, but the difference compared to those of the placebo was not significant. Systemic adverse events were not reported.

The first EPIT peanut study [22] confirmed the safety data of the previous study on cow milk allergies. This study was performed in a non-homogeneous population of children and adults using three different doses in a short period of 2 weeks. It showed that adverse events were more frequent with the high doses, but the differences were not significant.

The second peanut multinational double-blind study [23] was performed in a large population. The patients were randomized into four groups, and a significant difference in the response rate versus the placebo was shown in only the 250 µg treated group and was more evident in patients aged < 12 years, who achieved a > tenfold increase in the eliciting dose. As reported by the authors, these significant results are affected by some limitations: the small number of patients included when the division into the subgroups was performed, which evidences a possible relation with age; the primary endpoint not being stringent enough in the definition of clinical improvement; the exclusion of patients with severe anaphylaxis; and the different cutaneous sites of patch application, which might condition the allergen diffusion into the epidermidis. Additionally, the age, sex and race of the patients varied [20].

Despite these limits, in the following 2 years of this open study, the response rate increased to 63.3% after 12 months in < 12 years old patients and to 68.4% after 24 months, showing the progressive increase in efficacy, which is correlated with the duration of treatment and is associated with a significant reduction of adverse events. In the last EPIT study [24], in accordance with the previous trial, the authors underlined that EPIT is safe in children and that its efficacy is more evident in younger people. A decrease in specific IgE and a significant increase in IgG4 supports these encouraging clinical results. In the last two studies [23, 24], local adverse events were more frequently shown in the 250 µg treated patients, but this difference compared to the placebo was not significant. Systemic adverse events were rare, and a high rate of adherence was observed.

## Conclusions

The four clinical trials on EPIT for grass pollen allergic rhino-conjunctivitis have shown efficacy, safety and high adherence of this new route of AIT administration, and they were performed with different methods and evaluated by a VAS. However, for these reasons, validation by further studies performed according to EAACI and other international AIT guidelines are needed. The time of patch application ranging from 48 to 8 h in the last two studies of Senti et al. is very different from what is suggested by the kinetic study in an animal model [16]. A protocol taking into account the knowledge obtained from this model could be very useful to definitively identify a safe and effective allergen dose and the best time of patch application on the skin. Moreover, the cumulative dose might be reached through an increased number of patch applications in a longer period of 6 or 12 months or even 3 years, likely for SCIT and SLIT, with a possible definitive efficacy, long-lasting effect and same safety shown by EPIT in patients affected by allergic respiratory diseases. Studies performed in animal models [25, 26] show that EPIT, differently from SCIT and SLIT, may induce the production of naïve regulatory T-cells bearing a homing receptor for low respiratory airways as well. This activation of lung homing receptors, if it is confirmed in humans, might suggest the specific role of EPIT in the treatment of allergic asthma.

In FA, after the pilot study in children allergic to cow milk, peanut allergy studies showed a more favourable safety and elevated adherence compared to oral and sublingual AIT. In fact, more than 20% of drop-outs due to severe adverse reactions are reported in oral AIT, and a low adherence is achieved in SLIT trials [27]. Furthermore, eosinophil oesophagitis can be induced by these two types of immunotherapy [28], while according to EPIT studies on animal models [29, 30], this new allergen route of delivery may be specifically designed in cases of allergic patients with this disease. EPIT studies on cow-milk allergic children affected by oesophagitis are in progress [3]. The second and third studies in peanut allergic patients showed a significant EPIT efficacy, lower than that of oral AIT and similar to that of SLIT. However, further double-blind, placebo-controlled trials to support the open trial are needed. The third study confirms the previous results, especially in children aged less than 12 years. Moreover, the results obtained with a low dose and after a relatively short period of time (52 weeks) for its high safety and adherence suggest the possible efficacy in allergic children under 5 years as well. In younger children, where food allergen sensitization is starting to develop, EPIT could be safely given to induce a condition of tolerance without inducing the side effects and dropouts observed in oral AIT, which may be subsequently introduced to enhance the process of desensitisation. This protocol may be specifically indicated in those children who do not tolerate the first phase of oral immunotherapy. Another point that can be considered as underlined also by Wang and Sampson [31] might be to design a study where an increase in the surface of patch application is taken into account to allow for a higher allergen dose administration while avoiding side effects.

In conclusion, EPIT seems effective and safe in rhino-conjunctivitis and food allergies, although several open questions (i.e., dose, time of treatment, type of antigen, placebo effect) highlight the need of further studies.

## Abbreviations

AIT: allergen immunotherapy
APCs: antigen presenting cells
AR: allergic rhinitis
CPT: conjunctival provocation test
CRD: cumulative reactive dose
CTD: cumulative tolerated dose
EAACI: European Academy of Allergy and Clinical Immunology
EPIT: epicutaneous immunotherapy
FA: food allergies
NPT: nasal provocation test
OFC: oral food challenge
SCIT: subcutaneous immunotherapy
SLIT: sublingual immunotherapy
SPT: skin prick test
VAS: visual analog scale
